# Scalable Hydrogel Nanoparticle Enrichment for Enhanced Mass Spectrometry-based Proteomic Analysis of Plasma or Volumetric Dried Blood Samples

**DOI:** 10.21203/rs.3.rs-11053241/v1

**Published:** 2026-10-06

**Authors:** Amanda Campbell, Lindsey Becker, Simion Kreimer, Mitzi Gonzales, Koen Raedschelders, Jennifer Van Eyk

**Affiliations:** Odense University Hospital; Cedars-Sinai Medical Center; Cedars-Sinai Medical Center; Cedars-Sinai Medical Center; Cedars-Sinai Medical Center; Cedars-Sinai Medical Center

**Keywords:** Clinical proteomics, Mass Spectrometry, nanoparticle enrichment, dried blood, Plasma proteomics

## Abstract

Discovery-based proteomics for translational research requires workflows that reveal deep proteomes with a high degree of analytical precision, are scalable across large sample cohorts, and that can be adapted to different sample matrices. In our work, these sample matrices often extend beyond plasma to include volumetric dried blood samples (VDBS) that offer a minimally invasive alternative to standard venipuncture and enable remote, self-directed sampling. We describe the development and validation of a one-day, 96-well plate-based workflow built on the Ceres Nanotrap Protein Enrichment Affinity (PEAK) nanoparticle platform, optimized independently for both plasma and VDBS ahead of liquid chromatography–tandem mass spectrometry (LC-MS/MS). We benchmarked a tube-based Ceres workflow against both a Seer-encrichment and an unenriched SP3 workflow, assessed its suitability for VDBS, and translated this workflow to a plate-based protocol with a 2-hour digestion. The adapted workflow demonstrated robust reproducibility for both matrices: plasma yielded 4,937 unique proteins with a median inter-day CV of 11% whereas VDBS yielded 3,613 proteins with a median inter-day CV of 19%. Plasma and VDBS proteomes were highly concordant, with 75% of shared proteins quantified within one order of magnitude between matrices. We preliminarily evaluated the biological utility of this platform against a curated panel of 20 Alzheimer's Disease–associated proteins. Our results reveal the Ceres PEAK platform as a flexible, laboratory-adaptable workflow capable of deep and reproducible proteomic profiling of both plasma and VDBS.

## 1. Introduction

The total number of identified proteins is routinely used as a principle measure of assay performance in discovery-based proteomics. Identified proteins can reveal insights into pathology and can serve as prognostic or diagnostic biomarkers that may guide treatment, understand risk, and aid in patient stratification [1]. A critical and often underappreciated challenge in translational research extends beyond the identification of proteins and relates instead to the precision with which these proteins are quantified. Identifying large numbers of proteins in the absence of adequate precision, represented by percent coefficient of variation (%CV), results in a level of interpretive uncertainty that fundamentally limits the utility of discovery-based proteomic data.

The circulating proteome, identified and quantified from whole blood or its derivatives, is proposed to reflect physiological states such as aging and disease; its analysis can serve as a lens through which to view a patient [2, 3, 53]. The circulating proteome is routinely accessed using venous blood sampling, which, while routine, does require a trained phlebotomist, access to centrifugation, and cold-chain transport and storage [5]. By contrast, volumetric dried blood sampling (VDBS) devices, which utilize an absorptive component to accurately collect a predetermined volume of whole blood, rely on the stability of dried blood proteins to allow storage of samples at ambient temperature for several weeks [6, 7, 45, 4]. These features of VDBS enable study participants to collect their own blood samples remotely using a simple lancet [8, 9, 10]. As such, remote sampling can reduce barriers for inclusion in clinical trials, enable longitudinal monitoring of biomarkers or drugs, and help to understand the course of chronic diseases at the level of individuals [5].

The circulating proteome is notoriously complex, containing proteins estimated to number beyond ten thousand with a dynamic range that spans over 12 orders of magnitude. Highly abundant plasma proteins such as albumin and immunoglobulins, along with hemoglobin and red blood cell proteins for VDBS samples, can easily overwhelm the detection and quantitation of those lower abundant proteins that may be highly relevant and clinically informative [13, 14]. This inherent complexity renders the challenge of performing discovery-based proteomics with adequate analytical precision especially acute for circulation biofluids, and this challenge is common to all analytical platforms including mass spectrometry (MS) [11, 12].

In this study, we used the Ceres Nanotrap Protein Enrichment Affinity Kits (PEAK) [52] as a platform from which to develop an orthogonal nanoparticle enrichment workflow that is suited for routine, high-throughput analysis of the circulating proteome. Using the Seer Proteograph ONE [47] and SP3 [46] workflows as benchmarks, we initially surveyed the performance of two PEAK protocols for plasma. Next, we assessed these PEAK protocols for their compatibility with plasma and VDBS samples, and tailored the standard workflow to a plate-based, single-day protocol. Finally, we assessed the coverage, intra-assay reproducibility, and inter-day robustness of the modified assay for both plasma and VDBS samples. We conducted these studies with VDBS and plasma aliquots derived from patient samples obtained through an Alzheimer’s Disease research study. This design allowed us to correlate the quantified plasma and VDBS proteomes and to preliminarily evaluate the biological utility of this workflow to detect established or previously identified high value neurology and Alzheimer’s Disease-associated proteins.

## 2. Experimental Section

### 2.1 Patient Samples

A 5-mL K2-EDTA tube of venous blood (BD Vacutainer, NJ, USA) was obtained from two separate de-identified participants enrolled in the Alzheimer’s Disease and Related Dementias (ADRD) observational study at Cedars-Sinai Medical Center in November 2025. Samples derived from these blood draws are designated as patient A and B. Microsampling was simulated from the whole blood samples using 96 individual 10 μL VDBS devices (Neoteryx VAMS by Trajan, CA, USA), which were subsequently placed in an autorack (cat # 10101APK) and dried in the presence of desiccant and stored at room temperature for one week, akin to what is typical for routine VDBS collection. The remaining blood was centrifuged, and the resulting plasma was divided into 250 μL aliquots which were stored at −80°C until use. This study was institutional review board approved at Cedars-Sinai Medica Center (study approval identification: STUDY00003294) and conducted in accordance with the principles in the Declaration of Helsinki.

### 2.2 Sample Preparation

#### 2.2.1 Experimental Reagents

Chemicals were acquired from Sigma-Aldrich (MO, USA), except for dithiothreitol (DTT) and urea, which were obtained from Thermo Fisher Scientific (CA, USA). Iodoacetamide (IAA) was acquired from Spectrum Chemical (CA, USA). All solvents and reagents used for liquid chromatography tandem mass spectrometry (LC-MS/MS) were Optima-grade (Thermo Fisher), including trifluoroacetic acid (TFA) (Sigma-Aldrich). Sample preparation used 1.5 mL Protein LoBind tubes (Eppendorf, Germany) and were digested with MS-grade Trypsin-LysC mix (Promega, WI, USA). Nanotrap Protein Enrichment Affinity Kits were provided by Ceres Nanosciences (VA, USA).

#### 2.2.2 PEAK Nanoparticle Enrichment

Dried blood and plasma were prepared in accordance with the manufacturer’s protocol using a 16-well magnetic rack to pellet nanoparticles (Four E’s Scientific, NJ, USA). Proteins were extracted from the VDBS tips in 100 μL of either the Ceres Commercial Nanotrap Buffer 4, or the Ceres R&D buffer, for 30 min at 37°C with shaking. This difference in extraction distinguishes the Ceres Commercial Workflow from the Ceres R&D workflow. Afterwards, 50 μL supernatant or 50 μL plasma was diluted in 150 μL of either the Commercial or R&D buffer, and nanoparticles A, B, and C were added sequentially (66 μL each), followed by incubation for 30 min at room temperature (RT) with shaking. After two washes in 500 μL H_2_O, samples were resuspended in 100 μL 50 mM ammonium bicarbonate (ABC), 1 μL 500 mM tris(2-carboxyethyl)phosphine (TCEP) was added, and proteins were reduced for 30 min at 37°C. This was followed by alkylation with 5 μL 375 mM IAA for 30 min at RT in the dark. Samples were washed in 50 mM ABC followed by two H_2_O washes, then resuspended in 45 μL 40 mM ABC. Proteins were digested with the addition of 5 μg Trypsin/Lys-C (1 μg/μL) for 16–18 hrs at 37°C with shaking at 1,200 rpm. Digestion was terminated by the addition of 2 μL 5% TFA and the supernatant was transferred to a new 1.5 mL LoBind tube. Of note, one of three technical replicates prepared for the 2-hour digestion condition during the digestion time optimization experiment was excluded from analysis due to inadvertent incomplete addition of nanoparticle reagent, resulting in duplicate analysis (n = 2) for that condition.

The modified one-day, plate-based enrichment workflow was performed as described above, with the following modifications: Labware involved a 96-well plate magnet to pellet nanoparticles (Magnum EX, Alpaqua, MA, USA) and appropriately sized 8-channel pipettes. For dried blood, VDBS tips were added to a 96-well 500 μL plate (Axygen) and extracted with the R&D buffer on a thermoshaker. Either 50 μL supernatant from the VDBS extraction or 50 μL plasma was transferred into a new 0.8 mL 96 well plate (Abgene) and diluted with 150 μL Ceres R&D or Commercial buffer. Nanoparticles A, B, and C were added in successive 66 μL increments according to the Ceres PEAK protocol, followed by 30 min plate incubation at RT with agitation. Supernatant removal and washes were performed using an intermediate 500 μL plate (Axygen) that was placed on a second magnet to ensure that nanoparticles were not unintentionally discarded. The contents of the intermediate plate were also resuspended after each wash and transferred back to the original plate. The total volume of water used for washing remained 500 μL in keeping with the original protocol, but performed in two incremental additions that were ultimately combined: 300 μL for the intermediate plate, and 200 μL for the sample plate. Samples were resuspended in ABC, followed by TCEP and IAA additions with their respective incubations, as previously described. The washing procedure was repeated with ABC and water, followed by resuspension in 45 μL 40 mM ABC. Digestion was performed with 5 μg trypsin/Lys-C (Promega) for 2 hrs at 37°C with shaking at 1,200 rpm. Digestion was terminated by the addition of 2 μL 5% TFA, after which the supernatant was transferred to a Low Volume 96 well plate (Fisher Scientific Cat # 03-255-011) ahead of LC-MS/MS analysis.

#### 2.2.3 Unenriched Plasma – SP3

Neat plasma was prepared as previously with an automated Single-Pot, Solid-Phase-Enhanced Sample Preparation (SP3) procedure on a Biomek i7 Workstation (Beckman-Coulter, CA USA) [46]. Briefly, 5 μL of a 10X plasma dilution was combined with 35 μL lysis buffer (2% sodium dodecyl sulfate, 50 mM Tris·HCl) in a 96-well plate (0.5 mL, Axygen, CA, USA), 10 μL 200 mM dithiothreitol was added and the plate was incubated at 60°C for 30 min with shaking. Proteins were alkylated with 10 μL 400 mM IAA, followed by incubation for 15 min at RT in the dark. Next, 5 μL prepared magnetic beads were added (Silica Magnetic Beads, G-Biosciences, MO, USA) and acetonitrile (ACN) to a final concentration of 50%, followed by an 18 min off-magnet incubation and a 2 min on-magnet incubation at RT. Samples were subsequently washed twice with ethanol and twice with ACN. One μg Trypsin/Lys-C is added to each sample (0.02 μg/μL) followed by an overnight digestion (16–18hrs) at 37°C with shaking at 1,200 rpm. Proteins were eluted by the addition of 50 μL 4% dimethyl sulfoxide, followed by transferring the supernatant to a new plate (96-well PCR plate, BioRad, CA, USA) and then adding 1% FA to achieve a final concentration of 0.1% FA.

#### 2.2.4 Seer Nanoparticle Enrichment

Samples were prepared with a ProteographONE Assay Kit for 80 samples on an SP200 automated liquid handler according to the manufacturer’s protocol (Seer Inc., Redwood City, CA, USA). All reagents and labware were loaded onto the work deck, including the sample Transfer Plate containing 120 μL plasma per sample. Proteins were denatured and bound to nanoparticles, then reduced and alkylated. Peptides were digested with Trypsin/LysC for 2 hrs. Peptide yield was assessed with the Pierce Quantitative Fluorometric Peptide Assay (ThermoFisher Scientific), followed by vacuum centrifugation. Samples were stored dry at −80C, and resuspended in 0.1% FA to achieve a final concentration of 0.5 μg/μL based on the Fluorometric Peptide Assay results ahead of LC-MS/MS acquisition.

### 2.3 Liquid Chromatography Tandem Mass Spectrometry

All samples were stored at −80°C until analysis. Peptides were separated using a reversed-phase LC gradient on a Vanquish Neo LC system (Thermo Fisher Scientific) coupled to a 15 cm × 150 μm PepSep column packed with 1.5 μm C18 beads (Bruker) and a 170 nL Exp2 cartridge trapping column packed with PLRP particles (Optimize Technologies). The analytical column was kept at 45°C using heated sleeve. Samples were eluted over a 17-min linear gradient from 5% to 38% solvent B (80% ACN, 0.1% FA) in Solvent A (0.1% FA in water) at 1.2 μL/min followed by column washing and equilibration at 3.6 μL/min for a total of 20 min.

Data Independent Acquisition MS was performed using an Orbitrap Astral instrument (Thermo Scientific) operated in positive-ion mode with a nanospray ionization. The spray voltage was 2.3 kV, and the ion transfer tube temperature was 280°C. Full MS scans were acquired in the Orbitrap at a resolution of 240,000 over an *m/z* range of 360–1200, with an RF lens setting of 40%, a normalized Automatic Gain Control of 500%, and a maximum injection time of 10 msec. DIA scans were acquired in the Astral over an *m/z* range of 380–980 using automatically generated non-overlapping 4-*m/z* isolation windows. Fragment ions were detected in the Astral analyzer over an *m/z* range of 150–2000 using normalized HCD collision energy of 30%. The Astral scans used an RF lens setting of 40%, a normalized AGC target of 200% and a 3.6 msec maximum injection time.

### 2.4 Database Search

Raw files were searched using DIA-NN (v 2.3.1) [20] with an *in silico* spectral library using match-between-runs, trypsin/P digestion, and one missed cleavage allowed. The spectral library was predicted from the human SwissProt (20,412 human sequences, downloaded Aug 30th, 2024). Variable excision of N-terminal methionine and methionine oxidation were allowed, and all cysteines had fixed Carbamidomethylation. The MS1 and MS2 accuracy were set to 5 ppm and 10 ppm respectively, and the scan window was set to 10. All other settings were left at default, including retention time-dependent normalization and QuantUMS [48] quantification. The protein groups matrix generated by DIA-NN was used for statistical analysis. Protein isoforms were retained if they could be identified by at least 1 proteotypic peptide unique to the isoform.

### 2.5 Statistical Analysis

Statistical analysis was performed in Microsoft Excel and R (v 4.5.2) [22, 23]. Intra-day CVs were calculated by dividing the standard deviation with the mean for all replicates within one group prepared on the same experiment day (five per day). Inter-day CVs were calculated using daily mean and standard deviation values derived within each experimental group. For assessment of cross-matrix agreement, shared proteins detected in at least two replicates in either matrix were identified by matching protein groups between the plasma and VDBS datasets. Average protein intensities were log_2_-transformed, and the coefficient of determination (R^2^) was calculated from a linear regression of log_2_ VDBS intensity against log_2_ plasma intensity. Proteins were stratified by inter-day CV for visualization.

## 3. Results

### 3.1 Phase 1: Survey of two PEAK protocols for Plasma Proteomics

In the first phase of this study, we assessed the viability of two Ceres PEAK nanoparticle-based workflows for the enrichment and comprehensive MS-based proteomic analysis of plasma derived from a participant enrolled in an ADRD observational study. The two Ceres protocols differ only in the composition of an initial buffer for nanoparticle-protein interaction as well as extraction in the case of VDBS samples (“Nanotrap buffer 4” for the Commercial workflow, substituted by “R&D buffer” for the R&D workflow). The performance of these PEAK workflows was benchmarked against both the Seer ProteographONE nanoparticle enrichment platform and an unenriched plasma protocol utilizing SP3 beads. Each preparative method was assessed using five plasma aliquots, prepared in parallel. All prepared samples were acquired in a single LC-MS/MS batch to reduce between-batch LC-MS/MS variability. Platform performance was assessed using three primary metrics: total proteins identified, mean intra-assay CV, and the proportion of proteins meeting a CV < 20% precision threshold.

The Seer ProteographONE platform identified the largest number of proteins (6,977 total identifications), and achieved both the lowest mean intra-assay CV (8.6%) and the highest proportion of quantified proteins meeting the CV < 20% threshold (88%, 5,367 proteins detected in all five replicates below this threshold). The Ceres PEAK Commercial workflow identified 6,420 proteins (6,508 across all samples) at a mean intra-assay CV of 12%. 4,561 of these proteins were detected in all five Ceres PEAK Commercial replicates with CV < 20%, representing 78% of the total. The Ceres PEAK R&D workflow identified 5,598 proteins at a mean intra-assay CV of 16.2%, with 67% achieving CV < 20%. The SP3 workflow exhibited lower proteome coverage, identifying 2,268 proteins with a mean CV of 38%, and virtually no proteins quantified below the CV < 20% threshold (Fig. 1A–C). Comparison of overlapping proteins revealed that most identifications were shared between the Ceres Commercial and Seer workflows, with approximately 1,725 proteins common to all four workflows, roughly 2,000 proteins unique to Seer, and approximately 1,000 proteins unique to the Ceres Commercial workflow (Fig. 1A). Proteins quantified with high precision were distributed across the full dynamic range for both Seer and Ceres workflows (Fig. 1D), and the majority of proteins for all workflows were detected in all five replicates (Fig. 1E).

### 3.2 Phase 2: Performance of the Ceres PEAK Platform in Patient Plasma and Volumetrically Accurate Dried Blood

In the second phase of our study, we repeated our assessment of both Ceres PEAK workflows using an additional five plasma and five VDBS aliquots derived from the same patient blood draw. Samples were processed in parallel using the Ceres PEAK Commercial and R&D buffers and acquired in a single LC-MS/MS batch.

For plasma, the results were consistent with Phase 1: The Ceres PEAK Commercial buffer workflow identified 5,913 total proteins with a mean CV of 10.5%, and 84% of these proteins were detected in all five replicates with CV < 20%. The Ceres PEAK R&D buffer workflow identified 4,877 total plasma proteins with a mean CV of 14.2%, and 71% of these proteins achieved CV < 20% (Fig. 2A–C).

For VDBS samples, the relative performance of the two buffers was reversed compared to plasma. The Ceres PEAK R&D buffer workflow identified 4,513 proteins with a mean CV of 16.9%, 62% of which were detected in all five replicates at CV < 20%. By contrast, the Ceres PEAK Commercial buffer workflow identified 3,192 VDBS proteins with a higher mean CV of 19.2%, 53% of which were detected in all five replicates at CV < 20% (Fig. 2A–C). Despite the lower overall depth in VDBS relative to plasma, a large majority of detected proteins were shared regardless of matrix or buffer choice (Fig. 2A). The dynamic range of VDBS samples was lower than that of plasma, particularly with the Commercial buffer (Fig. 2D). The R&D buffer increased VDBS proteome coverage and reproducibility relative to the Commercial buffer (Fig. 2E).

### 3.3 Phase 3: Optimization of Digestion Conditions for a Single-Day Workflow

In the third phase of our study, we evaluated the impact of reduced digestion time, elevated temperature, and alternative digestion buffers on proteome coverage and analytical precision. Digestion duration was assessed by subjecting VDBS extracts from Patient A to 2-, 4-, and 6-hour incubations. Digestion buffer composition and temperature (37°C and 42°C) were subsequently evaluated in both plasma and VDBS extracts from a second patient (Patient B), using the appropriate Commercial and R&D buffers, respectively.

Proteome depth and intra-assay precision were comparable across the three digestion durations (Fig. 3A). The 2-, 4-, and 6-hour protocols identified 2,606, 2,612, and 2,584 proteins, respectively (Fig. 3A); note that the 2-hr condition was supported by duplicate analysis (n = 2) due to exclusion of one replicate in which insufficient nanoparticle reagent had been dispensed. The proportion of proteins meeting the CV < 20% threshold was 85% (2-hr) 68% (4-hr), and 78% (6-hr) (Fig. 3B) with median CVs of 7.3%, 14.7%, and 11.5% (Fig. 3C). We observed an increased missed cleavage rate from 11.5% in the 6-hr digestion to 12.5% when the digestion was decreased from the 6-hr to the 2-hr protocol, representing a difference of approximately one percentage point and remaining within the range previously observed with overnight digestions. MS1 signal, as well as identified proteins and peptides were also consistent across the tested digestion times (Fig. 3D).

Transfer of the 2-hour protocol to a 96-well plate format and evaluation of digestion conditions revealed that buffer composition and temperature had a modest overall effect. In plasma, temperature did not produce a clear difference in total protein identifications. For VDBS, a slight decrease in proteome depth was observed at 42°C compared to 37°C. Missed cleavage rates were highest with the Promega buffer (13 – 14%) and lowest with the Tris·HCl + CaCl_2_ buffer (10 – 12%), with variation within triplicates of the same condition (Fig. 3E). Based on these findings, a 2-hour Trypsin/LysC digestion at 37°C with shaking at 1,000 rpm using Tris·HCl + CaCl_2_ buffer was carried forward for the plate-based workflow.

### 3.4 Phase 4: Validation of the Plate-Based Single-Day Workflow

In the fourth phase of our study, we validated the optimized plate-based, single-day protocol by preparing five independent plasma and VDBS aliquots from Patient B on three separate days (n = 15 aliquots per matrix; n = 30 total). Plasma was processed with the Ceres PEAK Commercial buffer and VDBS with the R&D buffer, both with 2-hour Trypsin/LysC digestion at 37°C.

Plasma samples demonstrated consistent proteome coverage across the three days, with 4,908, 4,915, and 4,898 proteins observed on day 1, 2, and 3, respectively, (day 1 depicted in Fig. 4A). Inter-day CVs for plasma proteomes were 14.9% (day 1), 11.9% (day 2), and 14.4% (day 3) (Fig. 4B). Within each day, between 67 and 77%% of quantified plasma proteins had an intra-day CV ≤ 20% (Fig. 4C). A total of 4,530 unique proteins were identified in all 15 plasma replicates, of which 3,520 were detected in all 15 replicates with an inter-day CV < 20% (Fig. 4D).

VDBS samples also showed consistent protein numbers across the three preparation days, with 3,217 (day 1), 3,174 (day 2), and 3,372 (day 3) proteins observed with a majority of VDBS proteins also detected in plasma (day 1 data depicted in Fig. 4A). VDBS proteomes showed an average CV of 18.1% (day 1), 22.6% (day 2) and 20.7% (day 3) (Fig. 4B) Of 3,613 total unique VDBS proteins, 2,933 unique proteins were detected in each day, 54% of which reaching an inter-day CV < 20% (Fig. 4C). 2,080 proteins were detected in all 15 replicates across the three days (Fig. 4D). Median inter-day CV for VDBS was 19% (Fig. 4B). Intra-day CVs indicated that approximately 50% of quantified VDBS proteins had intra-day CV ≤ 20% on each day (Fig. 4C). Near-complete proteome overlap was observed across all three days in plasma, and a high degree of overlap was maintained in VDBS (Fig. 4E). Consistent with previous experiments, plasma showed a wider dynamic range than VDBS, and proteins with CV < 20 were spread throughout this range for both matrices (Fig. 4F).

To assess cross-matrix agreement, the median protein intensities from matched plasma and VDBS aliquots were compared for all proteins detected in at least two replicates per matrix. A total of 3,546 proteins were identified in both plasma and VDBS matrices (Fig. 5). Protein abundances were generally lower in VDBS than in plasma, and 2,678 of 3,546 shared proteins (75%) were quantified within one order of magnitude between the two sample types. The Pearson correlation coefficient between plasma and VDBS intensities was 0.662 for proteins with inter-day CV < 20% in plasma. This correlation was maintained for proteins with CVs above 20%. Several of the proteins showing the greatest relative enrichment in VDBS, including catalase, carbonic anhydrase 2 and 3, and multiple hemoglobin chains, are well-characterized components of the erythrocyte proteome.

### 3.5 Biological Relevance: Detection of Alzheimer's Disease-Associated Proteins

To assess the biological utility of the optimized plate-based workflow, we evaluated the detection and quantitative performance of a curated panel of 20 proteins with established or proposed associations with ADRD, as identified from the published literature [49–51]. Each target protein was assessed in both plasma (Commercial buffer) and VDBS (R&D buffer) across all 15 replicates of the optimized plate-based results.

Of the 20 curated AD-relevant proteins, 17 were detected in at least one sample type. Eight proteins were quantified with high reliability (inter-day CV < 20%) in at least one of the two matrices across all replicates (Table 1). These included apolipoprotein E (APOE), amyloid-β precursor protein (APP), and α-synuclein (SNCA), each detected in both plasma and VDBS with low intra-assay variability. Six additional proteins — including components of the complement and coagulation cascades — were detected at substantially higher rates in VDBS than in plasma. Among VDBS proteins, SNCA ranked among the highest by median abundance. Three proteins from the curated list were not detected in either sample type (Table 1).

Table 1. Blood proteins relevant for Alzheimer’s disease detected with Ceres PEAK nanoparticle enrichment. Plasma was prepared with the Commercial buffer while VDBS was prepared with the R&D buffer. Plasma and VDBS samples were prepared in five replicates across three days.

## 4. Discussion

The primary objective of this study was to determine the extent to which the Ceres Nanotrap PEAK Kits could serve as a platform from which to develop a scalable, one-day preparative workflow ahead of LC-MS/MS, suitable for discovery-based proteomics in both plasma and VDBS. Within the context of translational research efforts in our group, discovery-based platforms for the analysis of the circulating proteome are most effective if they can satisfy several interconnected criteria: First, proteome profiling must twin comprehensive protein identification with analytical precision. Specifically, in order to ensure that downstream biological analyses are supported by reproducible quantitation, a high proportion of detected proteins should achieve a high degree of intra-assay quantitative precision (CV < 20%). Second, because translational research can involve large sample numbers, discovery-based proteomic workflows need to be efficient, scalable, and robust. In our research setting, this ideally translates to 96-well plate-based workflows that are compatible with automated liquid handling platforms, and that can be completed within one working day. Moreover, these workflows must yield highly reproducible proteomes across independent preparations (low inter-day CV). Finally, to be broadly and routinely adopted, these workflows should be as adaptable and cost-effective as possible. The Ceres Nanotrap PEAK workflow, evaluated here in Commercial and R&D configurations for plasma and VDBS respectively, represents a viable laboratory-built option.

In Phase 1 of our study, we used human plasma aliquots and benchmarked the standard Ceres PEAK workflow against the Seer ProteographONE and the SP3 workflows to provide important context for evaluating performance. The Seer ProteographONE, a fully automated, sample-to-peptide system, identified the greatest number of proteins (6,977) with the highest precision (mean CV 8.6%; 88% proteins < 20% CV), consistent with its optimization for high-throughput plasma proteomics. The Ceres PEAK Commercial workflow approached this performance, identifying 6,420 proteins, 78% of which met the CV < 20% threshold. A substantial portion of Ceres proteins were shared with the Seer workflow. In contrast, the SP3 workflow, which does not enrich low-abundance proteins, identified fewer than one-third the number of proteins and achieved substantially higher CVs, reinforcing that enrichment provides a large benefit for broad-coverage discovery proteomics in plasma samples. These findings established the Ceres PEAK Commercial protocol as a viable workflow for plasma proteomics, offering good performance with the flexibility of a manual, laboratory-customizable procedure.

A central and practically consequential finding of this study was the opposite buffer performance observed between plasma and VDBS: the Ceres PEAK Commercial buffer outperformed the R&D buffer in plasma, while the R&D buffer yielded better protein coverage and lower intra-assay variability for VDBS samples. This matrix-dependent behavior may reflect fundamental differences in protein accessibility in the two sample types. In plasma, proteins are already solubilized and available for immediate nanoparticle interaction; in VDBS, proteins exist within a dried blood matrix absorbed onto a solid sponge-like platform from which they first must be extracted and solubilized prior to nanoparticle capture. The Commercial buffer is better suited for the soluble plasma proteome while the R&D buffer formulation is better suited for protein release from the VDBS matrix of the Mitra Microsampling Devices from Trajan that were used in this study. While the two buffers did produce overlapping proteomes, our observation of broader proteomes when a suitable buffer was used further suggests that buffer composition actively influences protein-nanoparticle enrichment [31, 29].

The transition from overnight trypsin digestion to a 2-hour protocol introduced only marginal increases in missed cleavages without meaningful loss of proteome depth or precision. This finding, which was consistent across plasma and VDBS extracts, and across different digestion buffer formulations and temperatures, seems to indicate that digestion completeness is not a bottleneck under shortened workflow conditions. Although the 2-hour digestion condition in this optimization phase of our study was initially only supported by duplicate analysis, our results supported the suitability of this shortened digestion protocol for single-day sample processing. The subsequent optimized plate-based inter-day precision results further support its suitability. Notably, digestion buffer composition and temperature (37°C vs. 42°C) had very modest effects on proteome coverage relative to the variation within replicates of the same condition. We inferred that neither digestion buffer chemistry within the range tested, nor tested temperatures were important sources of variability. While a more comprehensive assessment could be warranted, the workflow appears to be sufficiently robust across digestion buffer choices to accommodate procedural deviations across operators and laboratories.

The three-day plate-based validation demonstrated that the optimized workflow can yield reproducible proteomes across independent sample preparation days, a prerequisite for application in longitudinal or multi-batch translational studies. The median inter-day CVs of 11% for plasma and 19% for VDBS compare favorably to published benchmarks for manual discovery proteomics workflows, and these results are in line with what has been reported for automated platforms [25]. More than 75% of quantified plasma proteins and approximately 54% of VDBS proteins satisfied both the completeness criterion (detected in all 15 replicates) and the CV < 20% inter-day precision threshold. The slight improvement in inter-day CV for plate-based VDBS compared to the tube-based Phase 2 assessment suggests that adopting plate-based processing with an intermediate plate to reduce the risk of nanoparticle loss during wash steps, may contribute to improved consistency. Full automation on a liquid handling workstation would be expected to further decrease operator-dependent pipetting variation and reduce technical variability and throughput [25, 31]. The positive correlation between plasma and VDBS protein intensities (Pearson r = 0.662) for proteins with CV < 20% in plasma suggests that VDBS samples, subjected to this workflow, can provide a biologically informative proteomic snapshot that substantially recapitulates the plasma proteome. Importantly, 75% of shared proteins were quantified within one order of magnitude between the two matrices, supporting their broad comparability for discovery purposes while acknowledging the need for matrix-specific calibration in any targeted follow-up assay.

The decreased protein IDs observed in VDBS relative to plasma is consistent with the dilution effects inherent to dried blood extraction and with the inclusion of erythrocytes and other cellular blood components that are absent in plasma. The enrichment of erythrocyte-associated proteins (hemoglobin chains, carbonic anhydrase 2 and 3, catalase) in VDBS relative to plasma along with their higher intensities was expected given the composition of whole blood, and are consistent with prior comparisons of dried blood and plasma proteomes using conventional sample preparation [33, 34].

The detection of key ADRD-associated proteins — including APOE, APP, and SNCA — with high analytical precision (CV < 20%) in both plasma and VDBS demonstrates that the optimized workflow is sensitive to proteins of neurological relevance across the dynamic range of the circulating proteome. APOE is the strongest genetic risk factor for late-onset Alzheimer’s Disease [44], and its reliable quantification in both matrices establishes a foundation for proteoform-aware studies of APOE isoform distribution. APP quantification by DIA MS reflects the abundance of the full-length precursor protein rather than bioactive amyloid-β peptide isoforms, but the significance of APP protein level in blood relative to the established amyloid-β 42/40 ratio remains unclear and is likely proteoform-dependent [42, 43]. SNCA was reliably quantified in both matrices and ranked among the highest-abundance proteins in VDBS, consistent with its known enrichment in erythrocytes and platelets [38]. The detection of six complement- and coagulation-associated proteins at substantially higher rates in VDBS than in plasma is consistent with platelet activation and red blood cell content in dried whole blood, which can enrich proteins otherwise diluted below detection in plasma. These proteins may represent VDBS-enriched biomarker candidates for inflammatory or coagulation-related aspects of neurodegeneration that are less accessible from plasma alone.

Several limitations of the current study warrant consideration. First, the biological samples were aliquoted replicates derived from only two research participants enrolled in an Alzheimer’s Disease research study, and the cohort size precludes any statistical generalization of the detected proteome or its clinical interpretation. However, in our experience, purchased pooled plasma consistently yields a smaller proteome, and it is difficult to obtain replicates of VDBS and plasma from the same source. We conducted our study using these two patient samples not to draw conclusions about AD pathology, but to yield sufficient VDBS and plasma replicates from the same source to both develop and to test the workflow in a biologically relevant context. Second, the DIA-based LC-MS/MS approach quantifies protein groups rather than proteoforms; bioactive species such as amyloid-β 42 and β 40 isoforms, or specific phosphorylation states of tau, are not resolved by this strategy [39]. Third, nanoparticle enrichment is a dynamic, competition-driven process in which the composition of the sample itself can impact the protein enrichment for each particle type. This can introduce systematic biases through the enrichment step itself, which are difficult to resolve and account for in the absence of internal standard controls [35]. The complementary nature of the Ceres PEAK nanoparticle series (particles A, B, and C) likely broadens proteome coverage beyond what would be covered if a single particle is used. However, the captured proteome is far from representative of the total plasma or VDBS proteome. We used the VDBS MITRA microsampling devices from Trajan Scientific. The reproducibility that we observed in the proteome from VDBS samples may be due to the volumetric nature of these devices, and do not necessarily extend to non-volumetric dried blood samples, such as those collected on filter paper. Finally, the MS gradient used in this study was 17 minutes, and while this was chosen with throughput in mind, and longer gradients may be expected to increase proteome depth.

In conclusion, the Ceres Nanotrap PEAK platform can be adapted for one-day, plate-based proteomic profiling of both plasma and VDBS samples. While the Seer ProteographONE platform achieved the best results in the key metrics of total proteins identified and the proportion of proteins with CV < 20% [53], the single-day plate-based Ceres Nanotrap PEAK workflow also yielded impressive results. By demonstrating high intra-day precision, robust inter-day reproducibility across three independent preparation days, and meaningful cross-matrix proteome concordance, this workflow meets the practical requirements for large-scale translational proteomics studies. The detection of clinically relevant ADRD-associated proteins with quantitative reliability in both plasma and VDBS supports the application of this approach in biomarker discovery and longitudinal monitoring contexts. Future work will focus on automation-based reproducibility improvements, expansion to larger and clinically annotated patient cohorts, and integration of targeted follow-up assays for candidate biomarkers identified in the discovery phase.

## Supplementary Material

This is a list of supplementary files associated with this preprint. Click to download.

- floatimage6.jpeg

Table 1 is available in the Supplementary Files section.

## Figures and Tables

**Figure 1:**
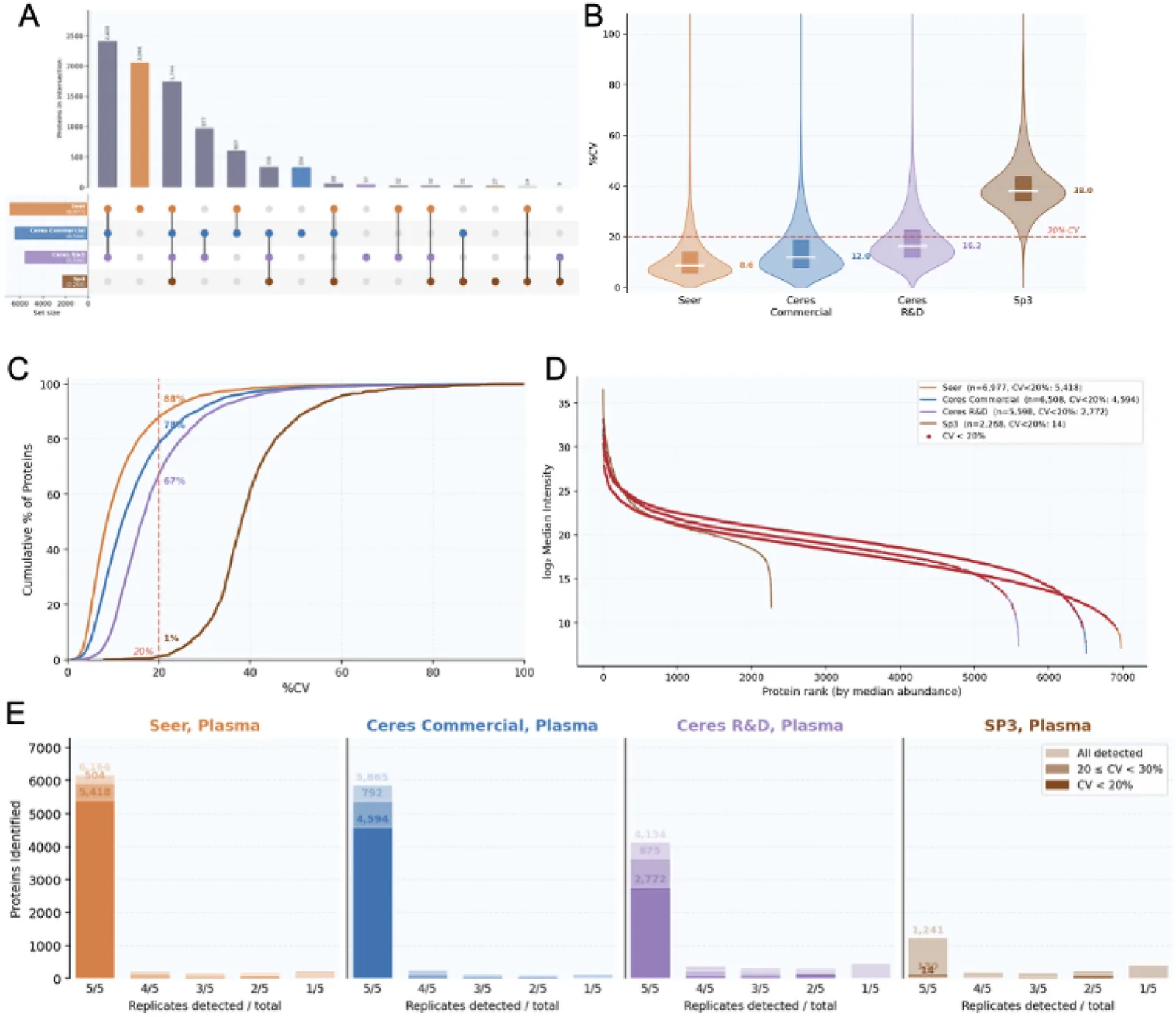
Survey of two Ceres sample preparation methods for plasma benchmarked against the Seer ProteographONE and an SP3 on-bead digestion method. Five aliquots of a plasma sample from Patient A were prepared for each of the protocols, and acquired in an LC-MS/MS batch. A) UpSet plot comparing shared proteins across the four methods. B) Intra-condition percent CVs from proteins detected in all five replicates within each method. Box plots mark the interquartile range and median; violin plots show the distribution; median CV annotated. C) Cumulative distribution of percent CVs. The red dotted line indicates the 20% CV threshold; the percentage of proteins below this threshold is annotated for each method. X-axis truncated at 100%. D) Dynamic range: log2 median intensity vs. protein rank; proteins with CV < 20% highlighted in red. E) Protein completeness for each method (Seer, Plasma; Ceres Commercial, Plasma; Ceres R&D, Plasma; SP3, Plasma). Proteins are grouped by the number of replicates in which they were detected. Color layers indicate all proteins identified (light), proteins with 20% <= CV < 30% (medium), and proteins with CV < 20% (dark); counts above the leftmost bar indicate totals in the all-replicate group. E) Bar plots of data completeness. For each method, proteins were grouped according to the number of replicates they were detected in. The percent CV of the proteins was also overlaid.

**Figure 2:**
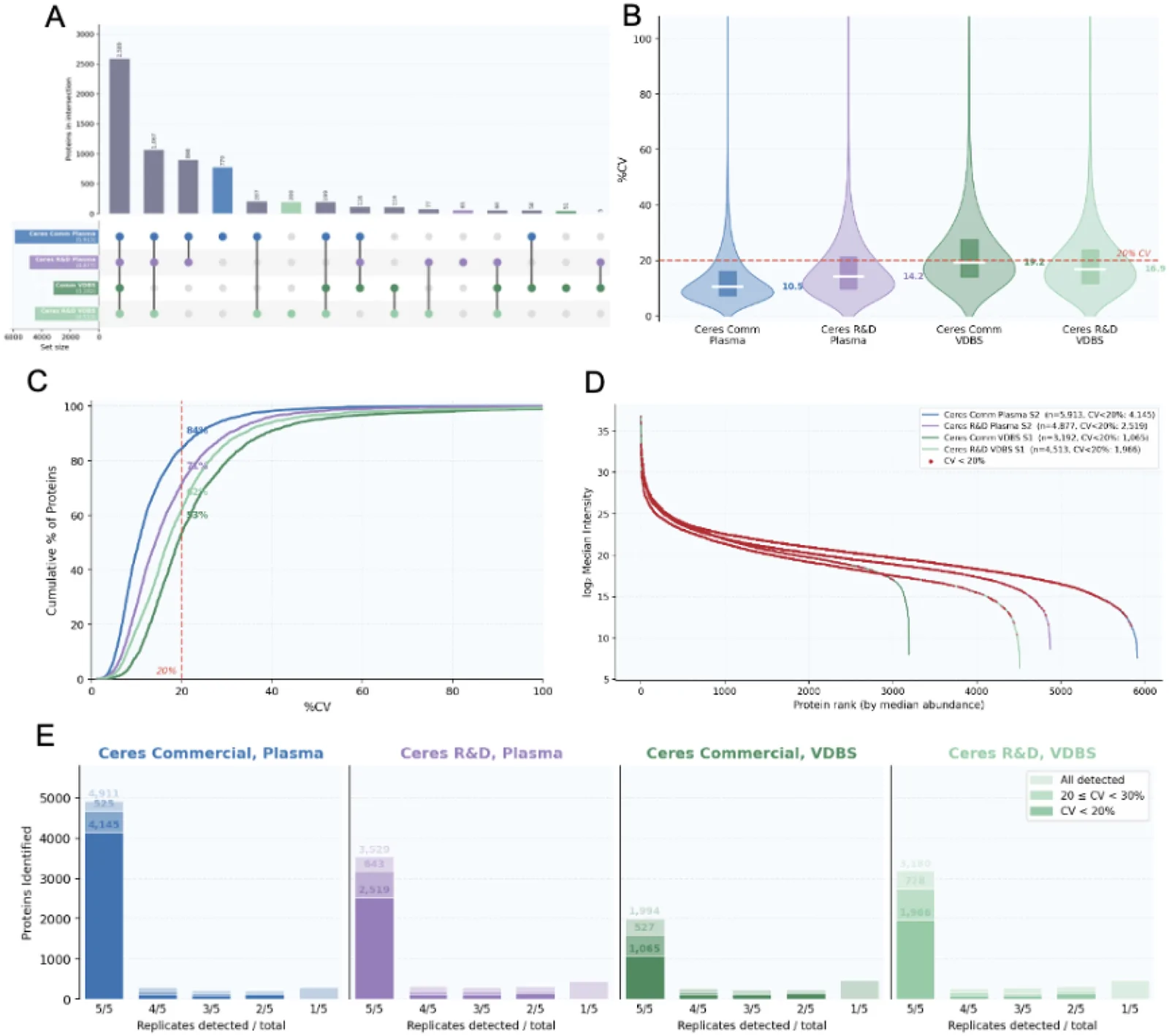
Initial Ceres PEAK nanoparticle assay evaluation. Five aliquots of plasma and five individual VDBS samples from patient A were prepared with each of two Ceres PEAK workflows, either the Commercial or R&D buffer. A) UpSet plot comparing shared proteins across each assay and biological matrix. B) Intra-condition percent CVs from proteins detected in all five replicates. Box plots mark the interquartile range and median; violin plots show the distribution; median CV annotated. C) Cumulative distribution of percent CVs. The red dotted line indicates the 20% CV threshold; the percentage of proteins below this threshold is annotated. X-axis truncated at 100%. D) Dynamic range: log2 median intensity vs. protein rank; proteins with CV < 20% highlighted in red. E) Protein completeness for four groups (Ceres Commercial, Plasma; Ceres R&D, Plasma; Ceres Commercial, VDBS; Ceres R&D, VDBS). Proteins are grouped by the number of replicates detected. Color layers indicate all proteins identified (light), proteins with 20% <= CV < 30% (medium), and proteins with CV < 20% (dark); counts above the leftmost bar indicate totals in the all-replicate group.

**Figure 3:**
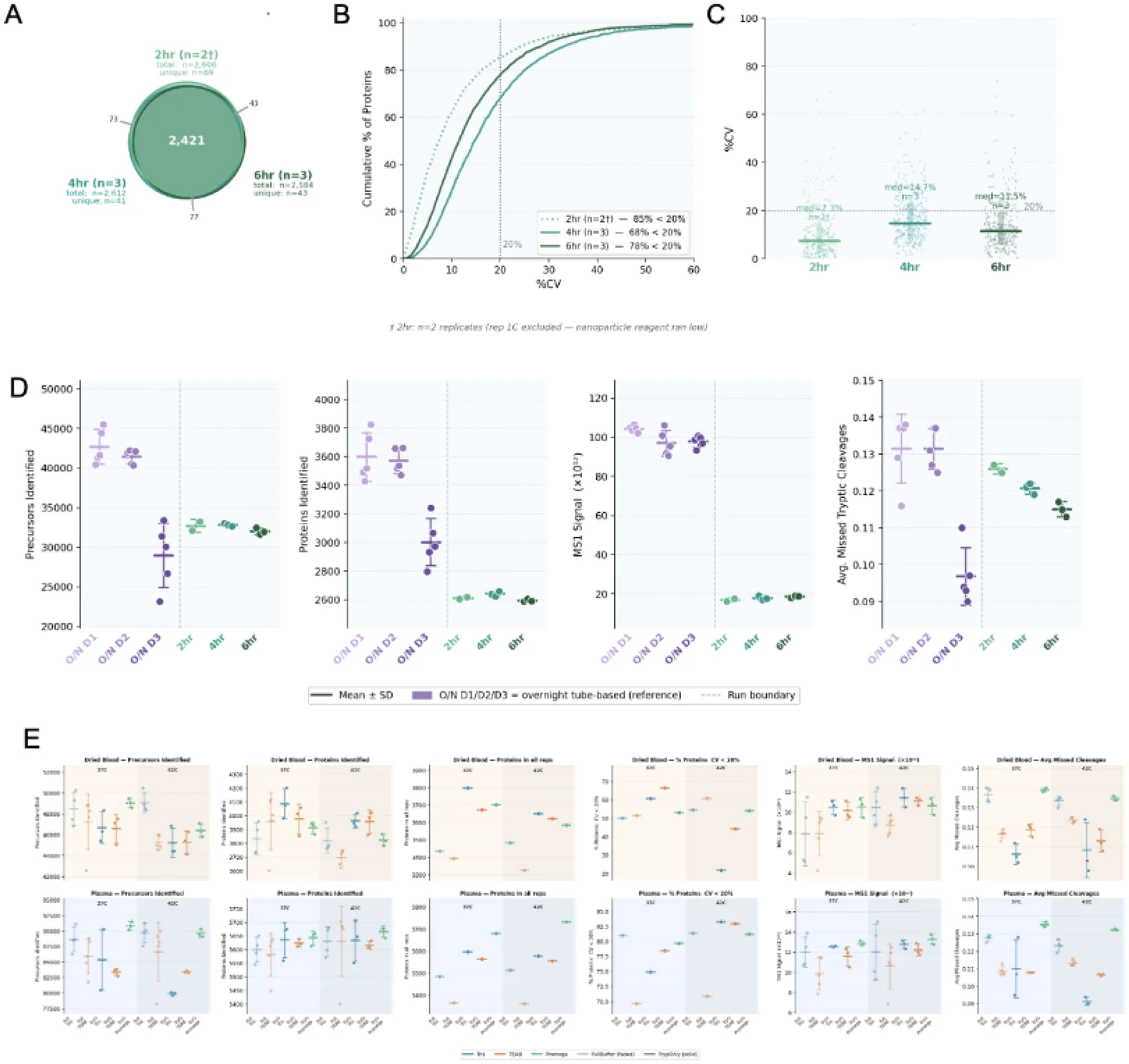
Optimization of digestion parameters for the plate-based Ceres PEAK assay. A-D) Key analytical parameters for VDBS samples (Patient A, tube-based format) across four digestion lengths (overnight, 2 hr, 4 hr, 6 hr). A) Proportional Venn diagram. B) Cumulative distribution of percent CVs. The red dotted line indicates the 20% CV threshold; the percentage of proteins below this threshold is annotated. X-axis truncated at 100%. C) Intra-condition percent CVs from proteins detected in all five replicates. The median CV annotated. D) precursors identified, proteins identified, total MS1 signal, and average percentage of peptides with >=1 missed cleavage. Five replicates were used for overnight digestion; three for 4 hr and 6 hr; two for 2 hr. The dotted line indicates samples not run consecutively. E) Optimization of buffer composition and digestion temperature for plasma and dried blood samples (Patient A, plate-based format). Tris, TEAB, and Promega buffers were tested at 37 degrees C and 42 degrees C. Six parameters are shown for each matrix: precursors identified, proteins identified, proteins detected in all replicates, proteins in all replicates with CV < 20%, total MS1 signal, and average percentage of peptides with >=1 missed cleavage. Abbreviations: CaCl2, calcium chloride; TEAB, tetraethylammonium bromide; Tris, tris(hydroxymethyl)aminomethane.

**Figure 4:**
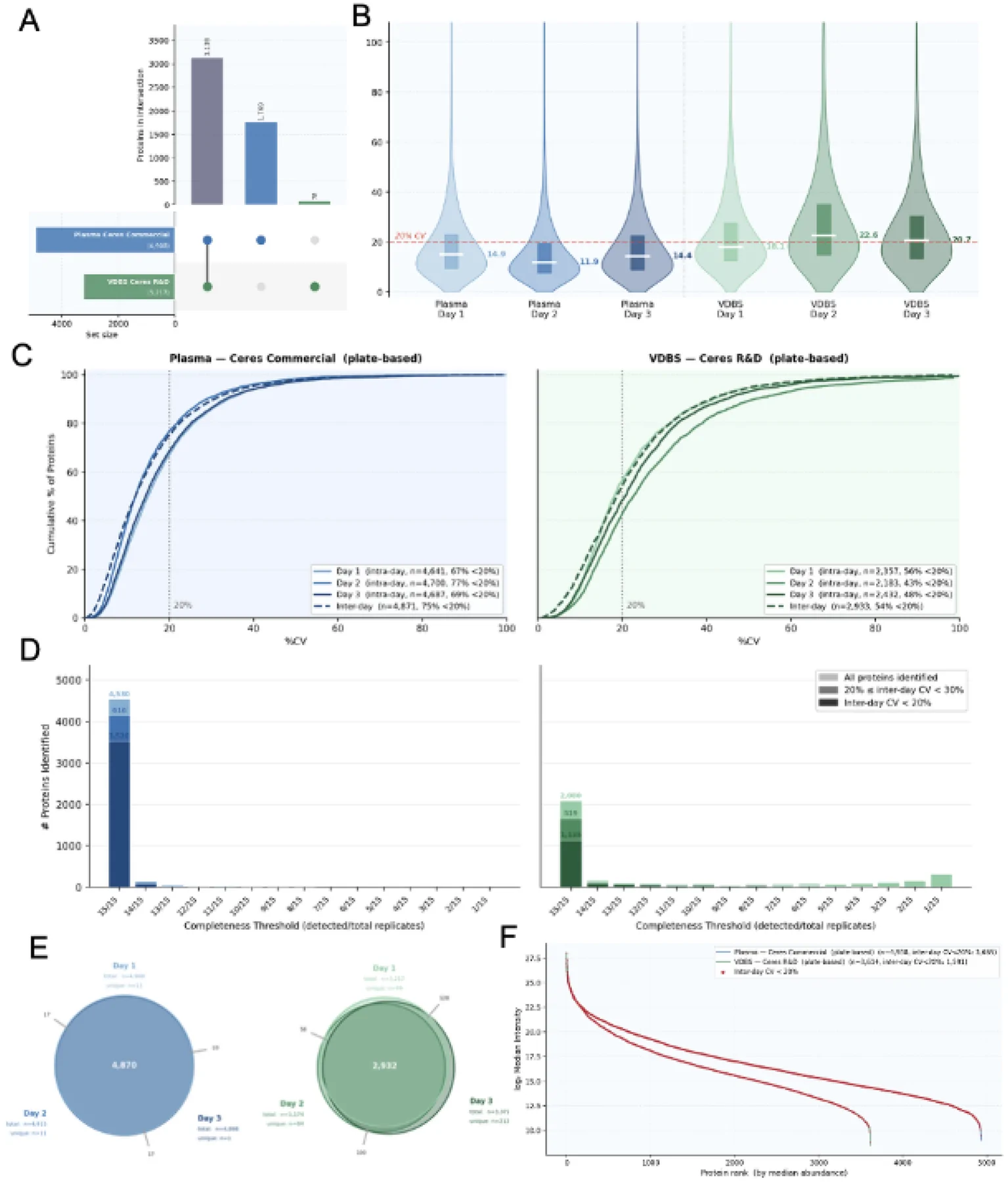
Evaluation of the optimized, plate-based Ceres PEAK assay for plasma and VDBS. Five plasma replicates from patient B were prepared with the Commercial buffer on each of three separate days, while five VDBS samples from the same patient were prepared with the R&D buffer, also on each of three separate days. A) UpSet plot comparing shared proteins across the two biological matrices. B) Intra-day percent CVs for proteins detected in all replicates for each day, shown for Plasma (Days 1–3; blue shades) and VDBS (Days 1–3; green shades). Box marks the interquartile range and median (white line); the 20% CV reference line is shown. C) Cumulative distribution of intra-day (solid lines, Days 1–3) and inter-day (dashed line) percent CVs for Ceres Commercial Plasma samples (left panel) and Ceres R&D VDBS samples (right panel). D) Protein completeness and inter-day reproducibility. Proteins are grouped by the number of replicates detected (out of 15 total), shown separately for Plasma (left) and VDBS (right). Color layers indicate all proteins identified (light), 20%≤ inter-day CV<30% (medium), and inter-day CV<20% (dark); counts above the leftmost bar indicate totals in the all-replicate group. E) Proportional Venn diagrams of inter-day protein identification overlap for Plasma (left) and VDBS (right). Each circle represents proteins identified on a given day; overlapping regions indicate shared proteins. F) Dynamic range: log2 median intensity ranked by protein abundance for Plasma and VDBS. Proteins with inter-day CV<20% are highlighted in red.

**Figure 5:**
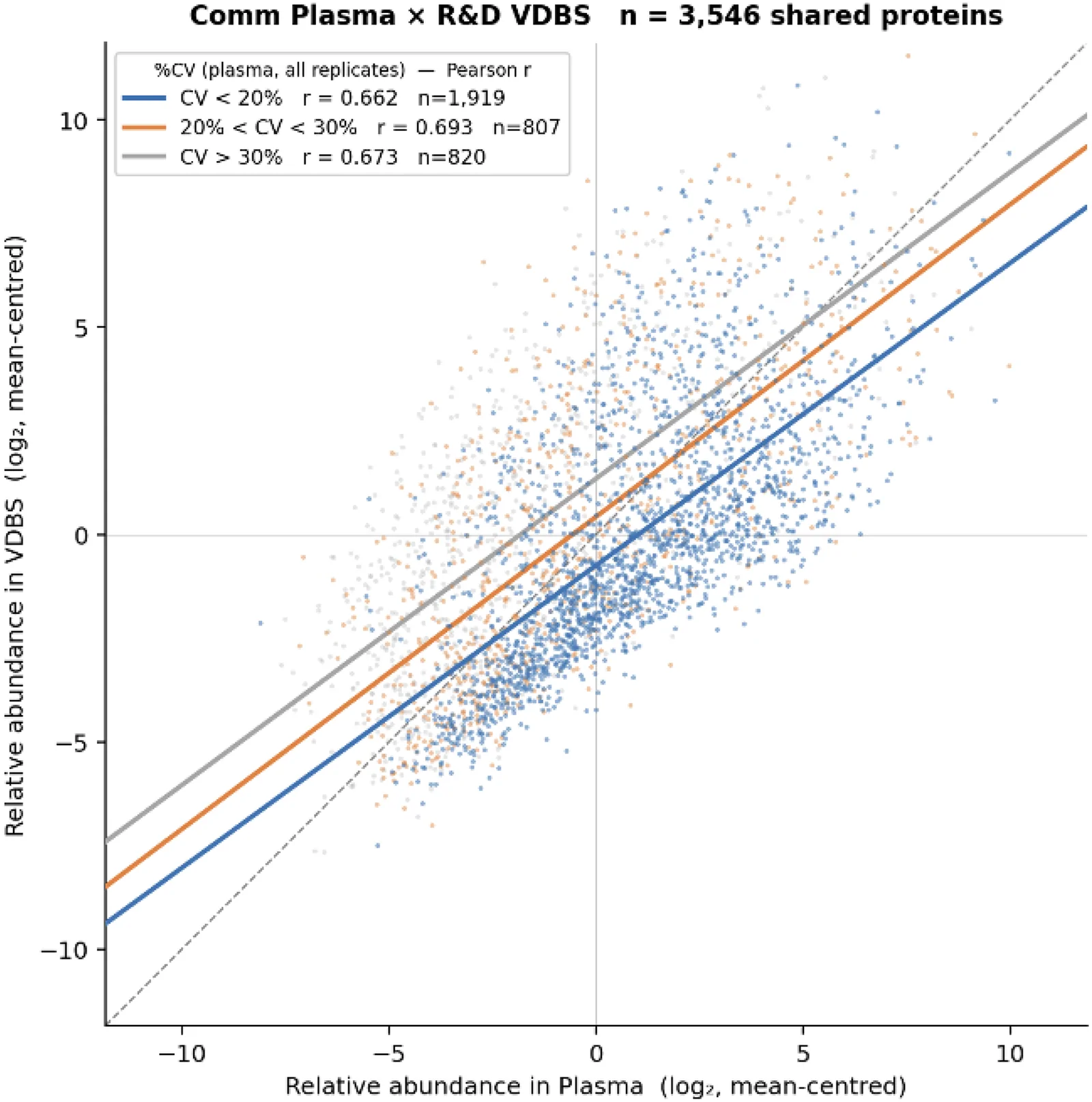
Agreement between protein abundances in VDBS and plasma. Results are derived from the optimized plate-based Ceres PEAK assay: plasma with Commercial buffer; VDBS with R&D buffer. Five replicates across three days for each matrix. Correlation between median log2 protein abundances in plasma and VDBS. Proteins were mean-centered and stratified by inter-day CV in plasma (CV<20%, 20%<CV<30%, CV>30%). Linear regression was performed separately per CV stratum; Pearson r and sample size (n) are indicated. Only proteins detected in at least two replicates per matrix were included.

## Data Availability

All mass spectrometry data will be deposited in ProteomeXchange via MassIVE under accession number MSV000103119. To view the dataset’s files, reviewers can log in to the MassIVE FTP server with this URL: ftp://MSV000103119@massive-ftp.ucsd.eduReviewers can access the data by using the following username for web access: MSV000103119_reviewer

## Abbreviations

ABC: ammonium bicarbonate
ACN: acetonitrile
AGC: automatic gain control
APOE: apolipoprotein E
APP: amyloid-β precursor protein
CV: coefficient of variation
DIA: data-independent acquisition
DTT: dithiothreitol
FA: formic acid
IAA: iodoacetamide
LC-MS/MS: liquid chromatography tandem mass spectrometry
MBR: match-between-runs
MS: mass spectrometry
RT: room temperature
TCEP: tris(2-carboxyethyl)phosphine
TFA: trifluoroacetic acid
VDBS: volumetric dried blood spots
α-synuclein: SNCA)
